# S2 Heart Sound Detects Aortic Valve Calcification Independent of Hemodynamic Changes in Mice

**DOI:** 10.3389/fcvm.2022.809301

**Published:** 2022-05-25

**Authors:** Valentina Dargam, Hooi Hooi Ng, Sana Nasim, Daniel Chaparro, Camila Iansen Irion, Suhas Rathna Seshadri, Armando Barreto, Zachary C. Danziger, Lina A. Shehadeh, Joshua D. Hutcheson

**Affiliations:** ^1^Department of Biomedical Engineering, Florida International University, Miami, FL, United States; ^2^Department of Human and Molecular Genetics, Florida International University, Miami, FL, United States; ^3^Interdisciplinary Stem Cell Institute, University of Miami Miller School of Medicine, Coral Gables, FL, United States; ^4^Department of Medical Education, University of Miami Miller School of Medicine, Coral Gables, FL, United States; ^5^Department of Electrical and Computer Engineering, Florida International University, Miami, FL, United States; ^6^Division of Cardiology, Department of Medicine, University of Miami Miller School of Medicine, Coral Gables, FL, United States; ^7^Biomolecular Sciences Institute, Florida International University, Miami, FL, United States

**Keywords:** aortic valve (AoV), calcific aortic valve disease, heart sounds classification, S2 heart sound, machine learning, chronic kidney disease

## Abstract

**Background:**

Calcific aortic valve disease (CAVD) is often undiagnosed in asymptomatic patients, especially in underserved populations. Although artificial intelligence has improved murmur detection in auscultation exams, murmur manifestation depends on hemodynamic factors that can be independent of aortic valve (AoV) calcium load and function. The aim of this study was to determine if the presence of AoV calcification directly influences the S2 heart sound.

**Methods:**

Adult C57BL/6J mice were assigned to the following 12-week-long diets: (1) Control group (*n* = 11) fed a normal chow, (2) Adenine group (*n* = 4) fed an adenine-supplemented diet to induce chronic kidney disease (CKD), and (3) Adenine + HP (*n* = 9) group fed the CKD diet for 6 weeks, then supplemented with high phosphate (HP) for another 6 weeks to induce AoV calcification. Phonocardiograms, echocardiogram-based valvular function, and AoV calcification were assessed at endpoint.

**Results:**

Mice on the Adenine + HP diet had detectable AoV calcification (9.28 ± 0.74% by volume). After segmentation and dimensionality reduction, S2 sounds were labeled based on the presence of disease: Healthy, CKD, or CKD + CAVD. The dataset (2,516 S2 sounds) was split subject-wise, and an ensemble learning-based algorithm was developed to classify S2 sound features. For external validation, the areas under the receiver operating characteristic curve of the algorithm to classify mice were 0.9940 for Healthy, 0.9717 for CKD, and 0.9593 for CKD + CAVD. The algorithm had a low misclassification performance of testing set S2 sounds (1.27% false positive, 1.99% false negative).

**Conclusion:**

Our ensemble learning-based algorithm demonstrated the feasibility of using the S2 sound to detect the presence of AoV calcification. The S2 sound can be used as a marker to identify AoV calcification independent of hemodynamic changes observed in echocardiography.

## Introduction

Calcific aortic valve disease (CAVD) is caused by calcium deposits in the aortic valve (AoV) and commonly leads to aortic stenosis, narrowing of the AoV that causes obstruction of blood flow and impaired cardiac function. Chronic kidney disease (CKD) patients are 9.0-fold more likely to develop AoV calcification and 2.7-fold more likely to develop aortic stenosis when compared to the general population (1, 2). Heart valve diseases in particular correlate with higher cardiovascular and all-cause mortality risks in dialysis patients compared to the general population (3, 4). The uremic toxins left in the blood due to poor filtration in CKD accelerate the progression of vascular and valvular calcification, as well as cardiac remodeling and fibrosis (4, 5). Due to a variety of overlapping risk factors and sociodemographic disparities, however, many CKD-induced cardiovascular diseases are underdiagnosed until end stages of disease (6–9).

Timely diagnosis of severe aortic stenosis has important implications in post-intervention outcomes, yet undetected asymptomatic CAVD affects ∼35% of the elderly population and is more common in low socioeconomic classes (10). Echocardiographic assessment of aortic stenosis in CKD patients, as with others, can be difficult and requires specific considerations due to extensive calcification of cardiac tissues, low-flow states, dialysis treatment, and masking of symptoms due to comorbidities (11). Heart valve diseases can be initially diagnosed by finding a murmur during in a cardiac auscultation exam, regardless of the presence of symptoms. The application of artificial intelligence on phonocardiogram (PCG) signals have significantly improved murmur detection and transformed the diagnostic value of the stethoscope (12–14). However, there are limitations in using murmurs to classify heart valve disease. Heart murmurs are typically benign or caused by high turbulent blood flow that succeed the structural remodeling changes of cardiovascular tissues. Only 30% of systolic murmurs in abnormal auscultatory findings are confirmed to be aortic stenosis by echocardiography (15, 16) and the intensity of an aortic stenosis murmur does not correlate well with severity of disease (17, 18).

New methods that can easily and non-invasively detect direct markers of heart valve disease independent of the presence of symptoms or blood flow changes are needed. According to the American Heart Association, the degree of calcification is a stronger predictor of clinical outcomes in patients with aortic stenosis compared to echocardiography (19). The (S2) heart sound results from AoV and pulmonary valve (PV) closure and, as a result, could be used as a direct measure of valvular structural properties. Changes resulting from pathological remodeling such as leaflet calcification at early stages of CAVD could alter S2 sound characteristics. Previous studies have reported CKD-induced CAVD using transgenic or nephrectomy-based mouse models (20–22). Tani et al. recently developed a mouse model, which induces CKD and subsequent vascular calcification using a high adenine-high phosphate diet without the need for invasive surgical procedures (23). This model showed significant vascular calcification; however, cardiac function and CAVD were not previously reported in these mice.

In this study, we employ this diet-induced CKD with vascular calcification model to determine whether changes in the S2 sound occur due to AoV remodeling. We assessed end-point AoV function using echocardiography and quantified AoV calcification post-mortem by imaging calcium mineral. Using an ensemble learning method, we trained an algorithm to predict the presence of AoV calcification using S2 sound features derived from principal component analysis (PCA). The threshold for labeling S2 sounds as either positive or negative for CAVD was determined by a positive calcium mineral signal in the AoV. After training, the algorithm was used to classify S2 sounds features from new mice as either Healthy, CKD without CAVD, and CKD-induced CAVD. The outcomes of this study suggest that S2 sound features can identify the presence of AoV calcification independent of hemodynamic changes that occur in CAVD.

## Materials and Methods

### Mouse Model of Chronic Kidney Disease

This study used eight-week-old male (*n* = 19) and female (*n* = 11) mice on C57BL/6J background. All mice were bred and housed on a 12:12-h day/night cycle at a room temperature of 20 ± 2°C in the Florida International University Animal Care Facility. The mice were given *ad libitum* access to food and water. All experimental procedures were approved by the Institutional Animal Care and Use Committee at Florida International University under protocol 20-006 and conformed to the Guide for the Care and Use of Laboratory Animals [National Institutes of Health (NIH), Bethesda, MD, United States] for scientific purposes. Body weight of the mice was recorded weekly using a digital scale.

**FIGURE 1:**
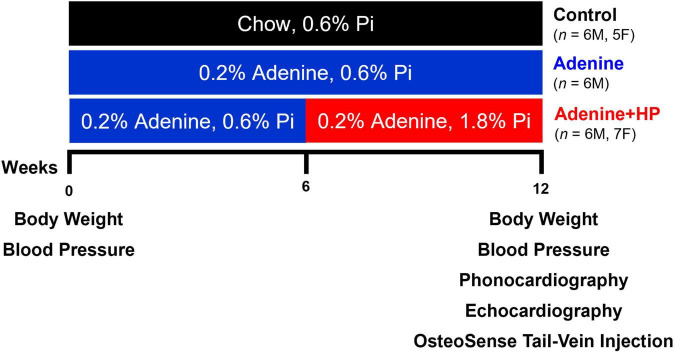
Experimental design. Study design for the induction of CKD and CKD-induced AoV calcification. Endpoint (Week 12) measures of AoV functional and remodeling parameters were assessed *via* echocardiography and quantification of OsteoSense signal. Phonocardiogram (PCG) signals were also recorded at endpoint.

Age-matched mice of both sexes were assigned to the following groups and diets: (1) Control group (6M, 5F) fed a normal chow diet for 12 weeks, (2) Adenine group (6M) fed the high (0.2%) adenine and normal (0.6%) phosphorous chow diet for 12 weeks to induce CKD, and (3) Adenine + HP group (7M, 6F) fed the high adenine and normal phosphorous diet for 6 weeks to induce CKD, followed by 6 weeks of a high adenine and high (1.8%) phosphorus (HP) diet to also induce cardiovascular calcification (Figure 1) (23). Mice on chow diet (0.6% total phosphorus) were used as negative controls. As per Tani et al., mice on the Adenine + HP group develop CKD-induced cardiovascular calcification starting at week 6 due to the HP-supplemented diet (23). The Adenine + HP group was used to study AoV calcification. Mice on the high adenine with normal phosphate diet were included to ensure that the changes in S2 were due to AoV calcification rather than CKD.

### Tail-Cuff Plethysmography

Mice were pre-warmed on a heating pad to 37°C for 5 min before recordings. Nocturnal blood pressure and heart rate were recorded during the animal’s dark cycle (between 7.00 p.m. and 6.59 a.m.) in isoflurane (iso 1–2%, oxygen 1–3%) anesthetized mice at week 0 (W0) and week 12 (W12) by tail-cuff plethysmography (CODA Monitor, Kent Scientific Corporation, Torrington, CT, United States). Ten measurements were taken from each mouse at the respective time-points, and data were averaged.

### Quantification of Aortic Valve Calcification

Two days prior to study end-point, the mice received a single tail vein injection of OsteoSense 680EX (80 nmol/kg, Perkin Elmer, Waltham, MA, United States), a fluorescent imaging agent that binds to calcium minerals (24). The AoV leaflets were fixed in 10% formalin at room temperature for 30 min. AoV leaflets from the non-coronary cusps (NCC), left coronary cusps (LCC), and right coronary cusps (RCC) were resected from the AoV after fixation. OsteoSense and autofluorescence (Excitation 488 nm, Emission 530 nm) signals were visualized in AoV leaflets with a 10× lens using a laser scanning confocal microscope (Olympus BX61, Center Valley, PA, United States). Z-stack images were generated using the Olympus FluoView version 4.2 software, and analyzed using a custom written MATLAB (MathWorks, Natick, MA, United States) script. Autofluorescence signal was used to obtain the total AoV leaflet volume whereas the OsteoSense signal was used to obtain the total AoV calcification volume. We converted the gray scale images obtained from the confocal into binary images using the same threshold for all samples. To quantify AoV calcification, we measured the total positive OsteoSense and autofluorescence signal for every Z-stack and calculated percentage of AoV leaflet calcification based on volume. Representative images of AoV leaflets were generated by the Z Project function in ImageJ (25).

### Phonocardiography

#### Data Collection

Phonocardiogram signals were recorded at W12 using a digital stethoscope (ONE, Thinklabs Medical LLC, Centennial, CO, United States). The stethoscope’s diaphragm diameter was reduced by using clear bumpers (12.7 mm Clear Bumper, Scotch Brand, St. Paul, MN, United States) to adjust for mouse heart size and reduce noise created by body movement. The mice were restrained in a supine position using surgical tape to minimize movement and anesthetized with isoflurane (iso 1–2%, oxygen 1–3%). Isoflurane to oxygen ratio was adjusted to visually ensure that the mouse’s abdomen movement, caused by the inhalant, was approximately once to twice per second. Abdomen movement per second is equivalent to breathing rate per second. PCG signals were acquired using MATLAB at 100K sampling frequency for 60–90 s.

#### Region of Interest – Sum-of-Squares Error Segmentation Method

**FIGURE 2:**
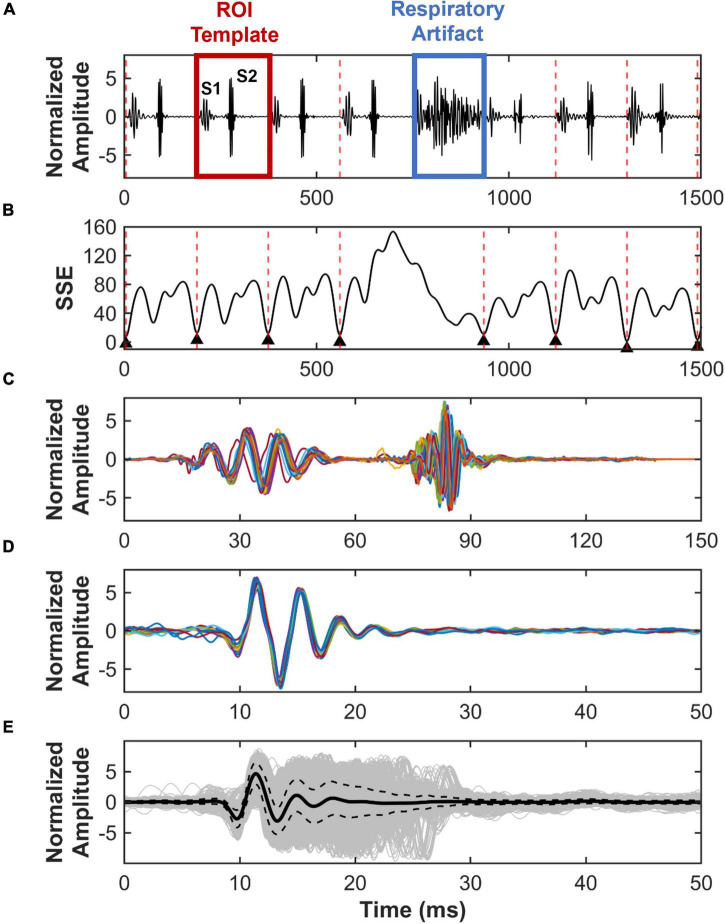
Region of interest – sum-of-squares error (ROI-SSE) segmentation method. Identification and segmentation of S2 sounds from recorded PCG signals. **(A)** Example of a normalized PCG signal recorded from a mouse, which includes S1 and S2 sounds, respiratory artifacts, and a selected ROI template for cycle segmentation. **(B)** SSE between ROI template and PCG recording, with the lowest error corresponding to the start of a new cardiac cycle. **(C)** Example of cardiac cycles segmented using ROI-SSE segmentation method. **(D)** Ten S2 sounds aligned in time. **(E)** In gray, superimposed S2 sound waveform of all mice with its average (solid black) and standard deviation (dotted black).

To analyze the S2 sound, we developed a segmentation method that finds all cardiac cycles in a recording. First, a region of interest (ROI) consisting of one cardiac cycle was manually selected per recording by identifying the start of systole and end of diastole (Figure 2A). The duration of systole and diastole was calculated to ensure that the selected ROI consisted of a cycle where systole precedes diastole, with the assumption that systole is shorter in duration than diastole. Once the ROI was correctly selected, the root-mean-squared (RMS) envelope of the ROI and the entire PCG recording was calculated. Then, the sum-of-squares error (SSE) was calculated between the envelope of the ROI and the envelope of PCG recording, using a temporal convolution kernel, where the ROI template moves through a sliding window of the PCG time domain signal. The locations with the lowest SSE (less than 10 and the closest to 0) correspond to the starting points of cardiac cycles, while the location of the highest error identify the location of a respiratory artifact (Figure 2B). An example of successful segmentation of several cardiac cycles using the ROI-SSE method is shown in Figure 2C. S2 sounds are then isolated and aligned in time to the start of the sound for further analysis. An example of 10 S2 sounds aligned in time is shown in Figure 2D.

#### Verification of Region of Interest – Sum-of-Squares Error Segmentation Method

To assess the performance of the ROI-SSE method, we compared it to an existing segmentation method. By simultaneously recording PCG and electrocardiogram (ECG) signals, the R-peak of the ECG signal can be used to mark the start of systole, which aligns with the start of the S1 sound (26). We simultaneously recorded PCG and ECG Lead-1 using 3 needle electrodes and a portable electrophysiology instrument (Lab Rat, Tucker Davis Technology, Alachua, FL, United States). Signals were collected from eight different mice for 60 s (sampling rate: 24,414 Hz). For each PCG recording, both the ECG and ROI-SSE segmentation methods were applied to find the number of cardiac cycles per recording and average cycle duration (milliseconds). The absolute error for the number of cycles and average cycle duration was calculated to depict the differences amongst methods.

#### S2 Sound Identification and Group Labeling

S2 sounds were downsampled (10K) and normalized prior to segmentation. S2 sounds from W12 were segmented using the ROI-SSE method and aligned in time to the start of the sound. The average S2 sound waveform from all sounds at W12 is shown in Figure 2E. To ensure that mice were labeled based on disease manifestation rather than the type of adenine and phosphorous diet combinations, we classified the S2 sounds based on presence or absence of AoV calcification. OsteoSense data were used to label S2 sounds from mice on the Adenine and Adenine + HP diets into two groups: (1) CKD + CAVD if the AoV OsteoSense signal greater than 0% and (2) CKD if the AoV OsteoSense signal was equal to 0%. The S2 sounds from Control mice were labeled as Healthy.

#### Algorithm Development

**FIGURE 3:**
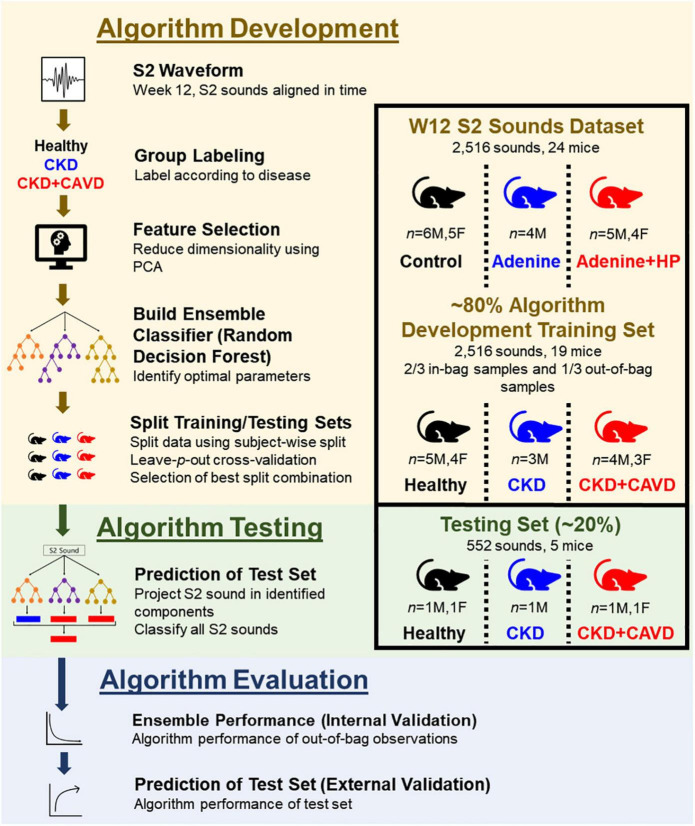
S2 heart sound classification algorithm. Development of algorithm to identify the presence of AoV calcification using the S2 sound in a mouse model of CKD-induced CAVD.

To determine if there were changes to the S2 sound due to CKD or CKD + CAVD, we developed an algorithm based on time domain PCA. A schematic of the algorithm is shown in Figure 3. To reduce the feature space dimensionality used for classification in subsequent steps, we omitted temporal modes identified by PCA that accounted for less than 5% of the variance in the within-session averaged S2 waveform records. We developed an algorithm based on classification trees and ensemble learning to identify if the S2 sound correlates to CAVD. Although classification trees are easy to build, they are unstable and have a high variance since they are dependent on the data selected for training (27). To mitigate this, we created an ensemble algorithm using the Random Forest (RF) bagging method that helps reduce the high variance. RF creates multiple decision trees and splits the training data into in-bag and out-of-bag samples. Then, it combines all tree predictions into one ensemble prediction and tests the performance of the model on the out-of-bag samples. Each tree was developed by randomly splitting the training data into in-bag (2/3 of training set S2 sounds) and out-of-bag (1/3 of training set S2 sounds) samples and random PCA features were used to split each node. The number of nodes for each tree matched the number of PCA features. A total of 60 trees were used for the ensemble model prediction, which was optimized based on the out-of-bag error. We used the features obtained from PCA as inputs for our algorithm to classify S2 sounds as: Healthy (mice fed normal chow), CKD, or CKD + CAVD.

#### Model Performance Based on Selection of Training/Testing Data

We used a subject-wise split approach to separate training and testing datasets to ensure that the algorithm can be tested on mice that were not included in the training set. For supervised learning models to accurately classify testing data, the training set must have a large sample size of subjects that is representative of a population (28). To mitigate for our low sample size, we applied a leave-*p*-out cross-validation (29) approach to split the data, where *p* represents every possible combination of mice in the testing data. Using this approach, we can find the best combination of mice (*p*) that ensures there are features representative of disease in the training set to accurately classify new mice in the testing set. Using an 80:20 split approach (*n* = 19 for training set, *n* = 5 for testing set) and accounting for both sexes in each group (Testing set: 1M, 1F Healthy, 1M CKD, 1M, 1F CKD + CAVD), there are 2,400 possible combinations (*p* = 2,400) of mice in the testing group. To assess performance of the model for all possible combinations, we report average accuracy and misclassification probability (1 – accuracy) for each experimental group.

After segmentation, each S2 sound was individually classified based on disease, with each mouse contributing multiple sounds that correlate to the same disease characteristic. Each S2 sound was classified based on presence of disease: 0 (Healthy), 1 (CKD), or 2 (CKD + CAVD). Subject-wise split ensures that the algorithm is identifying new cases of disease, separate from the ones used to train the algorithm. Therefore, our algorithm performance was based on the classification of each individual S2 sound rather than per subject. This approach increases the number of samples used to identify algorithm performance, since the testing set number of S2 sounds is much greater than the number if mice. By doing this, we can also test the feasibility of using a single S2 sound per subject to identify AoV calcification. S2 sounds from the testing set were projected on the components identified in the training set. The features obtained from PCA were used as inputs of the testing set for the algorithm.

#### Final Algorithm Performance

To test the hypothesis that the developed algorithm can use the S2 sound to classify and differentiate between Healthy, CKD, and CKD + HP mice, there must be a combination of mice (*p*) that can accurately classify all three groups. To determine the best algorithm performance, we summed the misclassification error for all three groups and selected the combination of mice with the lowest misclassification error. The selected combination of mice was used to test our hypothesis and reported as the final algorithm.

#### Final Algorithm Evaluation/Statistical Analysis

For internal validation, out-of-bag error and misclassification error for the out-of-bag samples were measured. For the testing set, we used the area under the curve (AUC) of the receiver operating characteristic (ROC) curve as a metric of algorithm performance. We reported the normalized confusion matrix to compare final algorithm predicted value (S2 sound classification from algorithm) vs. true value (classification based on post-mortem disease measures). Negative predictive value (NPV) and positive predictive value (PPV), for both CKD and CKD + CAVD, were quantified.

### Echocardiography

#### Data Collection

Cardiac and valvular hemodynamic parameters were evaluated using a Vevo 3100 imaging system (FUJIFILM VisualSonics, Toronto, ON, Canada) at the Sylvester Comprehensive Cancer Center, University of Miami. A day prior to the procedure, fur on the chest area was removed using depilatory cream. Echocardiography was performed following the protocol by Casaclang-Verzosa et al. (30) to evaluate valvular and cardiac function in mice. Prior to imaging, the mice were placed under a heating lamp to ensure normal body temperature (36.5–38°C). Mice were anesthetized using isoflurane (iso 1–2%, oxygen 1–3%). Inhalant anesthetic ratio was adjusted to maintain heart rate between 450 and 500 bpm and minimize respiratory artifacts due to deep anesthesia. Three ultrasound modalities were used to acquire images: B-mode, M-mode, and Doppler (pulsed wave and tissue). Transducer position was adjusted to acquire images of the valves and heart relative to long-axis view. Functional valvular parameters were obtained for the AoV and PV. Assessment of valvular function included both quantitative (e.g., transvalvular velocity and pressure) and qualitative (e.g., regurgitant jet) measures. Cardiac parameters were obtained to evaluate left ventricular (LV) function. Assessment of LV function included traditional quantitative measures such as ejection fraction and stroke volume.

#### Data Analysis

The echocardiogram data were analyzed with Vevo Lab 3.2.6 software (FUJIFILM VisualSonics, Toronto, ON, Canada). All measurements were obtained from three cardiac cycles and the average value was used for data analysis. For measures of volume, which include ejection fraction and stroke volume, parameters were obtained using the long-axis B-mode view. Wall size values were obtained from long-axis M-mode view.

### Statistical Procedures

Statistical analysis for all measures, excluding PCG signals, was performed using Prism version 6.0 (Graph Pad, La Jolla, CA, United States). One-way ANOVA with Tukey’s multiple comparison test was used to compare end-point parameters between the groups. A level of *P* < 0.05 was considered statistically significant. All results are presented as mean ± SEM, where *n* represents the number of mice per group.

## Results

### Chronic Kidney Disease Mouse Model Induces Aortic Valve Calcification

The survival rate per group was: 100% Control (*n* = 11/11), 66.7% Adenine (*n* = 4/6), and 84.6% Adenine + HP (*n* = 11/13). We excluded two of the females in Adenine + HP group because one had a bicuspid AoV and the other had severe LV calcification. Body weight was significantly reduced from W0 to W12 in mice from the Adenine and Adenine + HP groups (Table 1). Control mice gained an average of 4.87 ± 0.81 g from W0 to W12, while the Adenine and Adenine + HP mice lost an average of 6.79 ± 0.37 and 2.62 ± 0.54 g, respectively. Body weight of mice in the Adenine and Adenine + HP was statistically different from Control mice at W12. Heart rate of the Adenine group was statistically different than the Control group at both W0 and W12 (Table 1). There were no significant changes in mean arterial, systolic, or diastolic blood pressure amongst groups at either W0 or W12 when compared to Control group (Table 1). Every group had lower mean arterial, systolic, or diastolic blood pressure at W12 when compared to W0.

**TABLE 1:** Differences in systemic.

		Control	Adenine	Adenine + HP
Body weight (g)	W0	19.48 ± 1.12	20.55 ± 0.88	20.21 ± 1.16
	W12	24.35 ± 1.49^#^	13.76 ± 0.51***^###^	17.59 ± 0.62**
Heart rate (beats per minute)	W0	521.3 ± 3.7	468.6 ± 13.8**	497.5 ± 11.0
	W12	511.9 ± 11.8	446.8 ± 19.4*	483.3 ± 12.1
Mean arterial pressure (mmHg)	W0	116.8 ± 1.8	117.6 ± 3.2	122.1 ± 3.0
	W12	93.63 ± 2.85^####^	99.82 ± 4.47^#^	90.11 ± 2.34^####^
Systolic blood pressure (mmHg)	W0	130.4 ± 1.99	131.8 ± 3.61	136.2 ± 3.45
	W12	106.8 ± 3.18^####^	110.3 ± 4.92^#^	102.8 ± 2.35^####^
Diastolic blood pressure (mmHg)	W0	110.5 ± 1.77	111.0 ± 3.01	115.4 ± 2.88
	W12	87.56 ± 2.71^####^	95.12 ± 4.33^#^	84.22 ± 2.42^####^

Body weight, heart rate, and blood pressure measurements in Control, Adenine, and Adenine + HP mice. Values are expressed as mean ± SEM; *compare across groups vs. Control; ^#^compare within group vs. W0.

**P < 0.01 vs. Control group; ***P < 0.001 vs. Control group; ^###^P < 0.001 vs. W0 within the same group; ^####^P < 0.0001 vs. W0 within the same group.

**FIGURE 4:**
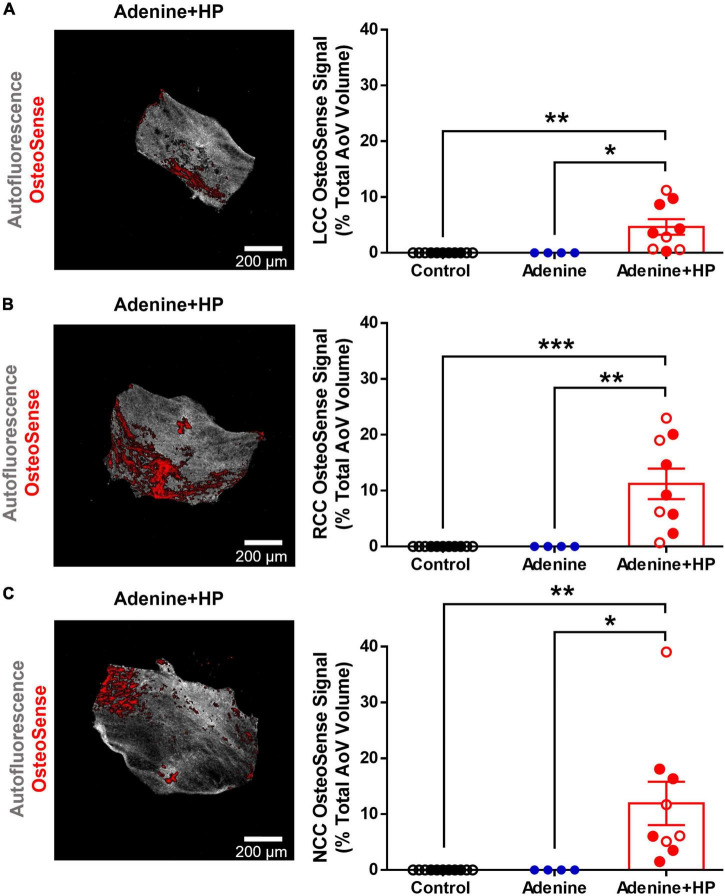
Aortic valve calcification. Image representation and quantification of AoV calcification expressed as percent positive of OsteoSense signal normalized to the total AoV volume. **(A)** Left-coronary cusp (LCC). **(B)** Right-coronary cusp (RCC). **(C)** Non-coronary cusp (NCC). One-way ANOVA with Tukey’s multiple comparisons test: *P < 0.05, **P < 0.01, ***P < 0.001. Male mice (●) and female mice (○).

We confirmed that only the Adenine + HP group had significant vascular calcification, as previously reported by Tani et al. (23). Similarly, only the mice on the Adenine + HP diet had positive OsteoSense signal in the AoV leaflets. AoV calcification was found near the base and belly regions of the Adenine + HP group leaflets, as shown in Figures 4A–C, with a total average calcification of 9.28 ± 0.74%. On average, the NCC was the most calcified leaflet (11.95 ± 3.88%), followed by the RCC (11.22 ± 2.73%) and LCC (4.66 ± 1.40%), however, differences amongst the three leaflets were not significant.

### Region of Interest – Sum-of-Squares Error Method Accurately Segments Heart Sounds

Using the same recording, the average time per cardiac cycle found with the ECG and ROI-SSE segmentation methods were 147.08 ± 1.80 and 146.70 ± 1.83 ms, respectively (*n* = 8). The absolute error between segmentation methods for the time per cardiac cycle was 0.004 ± 0.001 ms, which accounts for 0.00003% of the total average cycle time. The number of cycles identified using the ECG and ROI-SSE segmentation methods were 134.63 ± 2.25 and 126.88 ± 2.35, respectively, with an absolute error of 0.063 ± 0.007. On average, the ROI-SSE method identified 7.75 ± 1.16, or 5.78 ± 0.01%, fewer cycles than the ECG method.

### Cardiac Murmurs Can Be Detected in Mouse Phonocardiogram Signals

**FIGURE 5:**
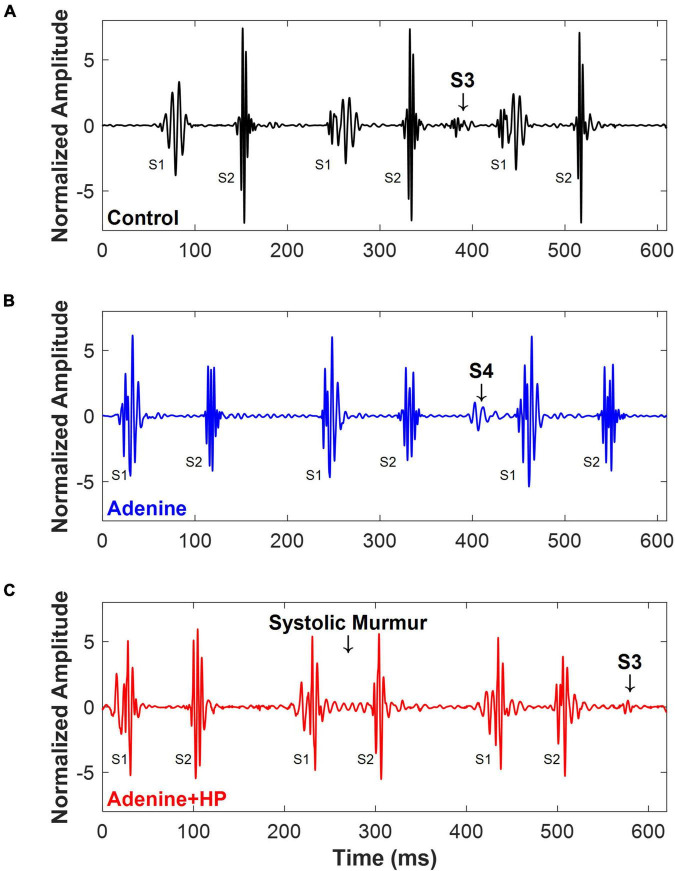
Cardiac murmurs in mice. Examples of cardiac murmurs detected in PCG signals of mice. **(A)** Control mice (*n* = 6/11) appeared to have an S3 sound. **(B)** Adenine mice (*n* = 3/4) appeared to have an S4 sound. **(C)** Adenine + HP mice appeared to have a systolic murmur (*n* = 7/9) and an S3 sound (*n* = 5/9).

Cardiac murmurs were observed in some of the PCG signals recorded (Figure 5). Some of the Control mice (*n* = 6/11) had an S3 sound found during diastole (Figure 5A). In the Adenine group, we found that some mice (*n* = 3/4) had an S4 sound during diastole (Figure 5B). A systolic murmur (*n* = 7/9), holosystolic in appearance, and an S3 sound (*n* = 5/9) were detected in some mice from the Adenine + HP group (Figure 5C). The murmurs and extra sound findings did not appear in each cardiac cycle, as shown in Figure 5. Systolic murmurs were detected in the Adenine + HP group, prevalent in 0.82% of all cardiac cycles. Diastolic murmurs, defined as the presence of an S3 or S4 sound, were found in the following percentage of all cardiac cycles per group: Control 1.10%, Adenine 3.94%, and Adenine + HP 0.87%.

### Model Performance Variance Based on Subject-Wise Split

**FIGURE 6:**
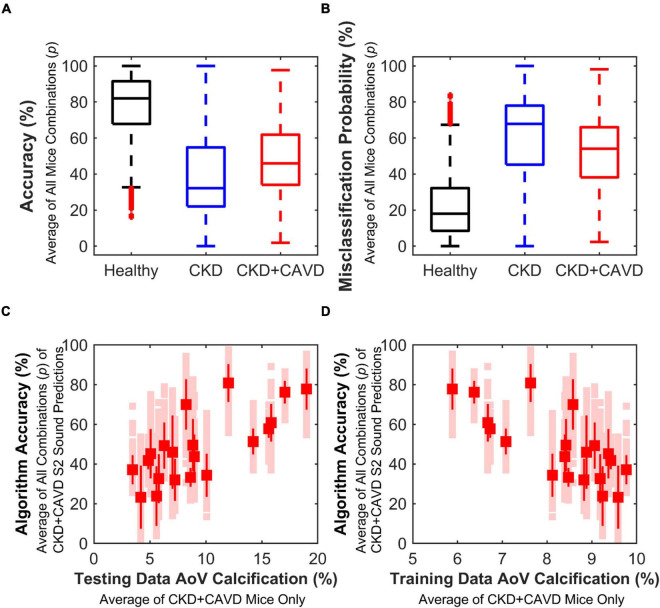
Performance based on leave-*p*-out cross-validation method. Variability of algorithm performance using leave-*p*-out cross-validation method, which tests performance based on different combinations of mice (*p* = 2,400) for training and testing sets. **(A)** Accuracy of all combinations. **(B)** Misclassification error of all combinations. **(C)** Correlation between algorithm accuracy and average testing set AoV calcification of CKD + CAVD mice. As testing set average AoV calcification of CKD + CAVD mice increases, accuracy of S2 sound classification increases (*r* = 0.73). **(D)** Correlation between algorithm accuracy and average training set AoV calcification of CKD + CAVD mice. As training set average AoV calcification of CKD + CAVD mice increases, accuracy of S2 sound classification decreases (*r* = –0.73).

Using a leave-*p*-out cross-validation method (29), we found that there were 2,400 different ways our mice could be split into the training and testing sets. The average number of S2 sounds in the training and testing sets for all possible combinations of mice (*p*) was as follows: Healthy 1,002:218 (±38), CKD 277:92 (±22), and CKD + CAVD 723:204 (±51). Keeping 20% of mice for testing data per group with the remaining 80% used as training, yielded 2,400 different combinations of mice (*p*). The mean accuracy (mean ± SD) for all combinations of mice was: Healthy 77.73 ± 17.91%, CKD 39.85 ± 26.06%, and CKD + CAVD 48.47 ± 20.25% (Figure 6A). The mean misclassification error (mean ± SD) for all combinations of mice was: Healthy 22.26 ± 17.91%, CKD 60.15 ± 26.06%, and CKD + CAVD 51.63 ± 20.25% (Figure 6B). To identify whether the amount of AoV calcification in the mice used for the training set affects classification accuracy of the testing set, we identified the average accuracy for all combinations of mice as a function of average AoV calcification in the training and testing sets (Figures 6C,D). There was a positive correlation (*r* = 0.73) between algorithm accuracy and average AoV calcification in the testing set (Figure 6C) and a negative correlation (*r* = −0.73) between algorithm accuracy and average AoV calcification in the training set and (Figure 6D). The combination of mice (*p*) that yielded the lowest sum of misclassification error for all groups (error = 28.51) was selected for our final algorithm.

### S2 Sound Characteristics Differ Due to Chronic Kidney Disease and Chronic Kidney Disease-Induced Aortic Valve Calcification

**FIGURE 7:**
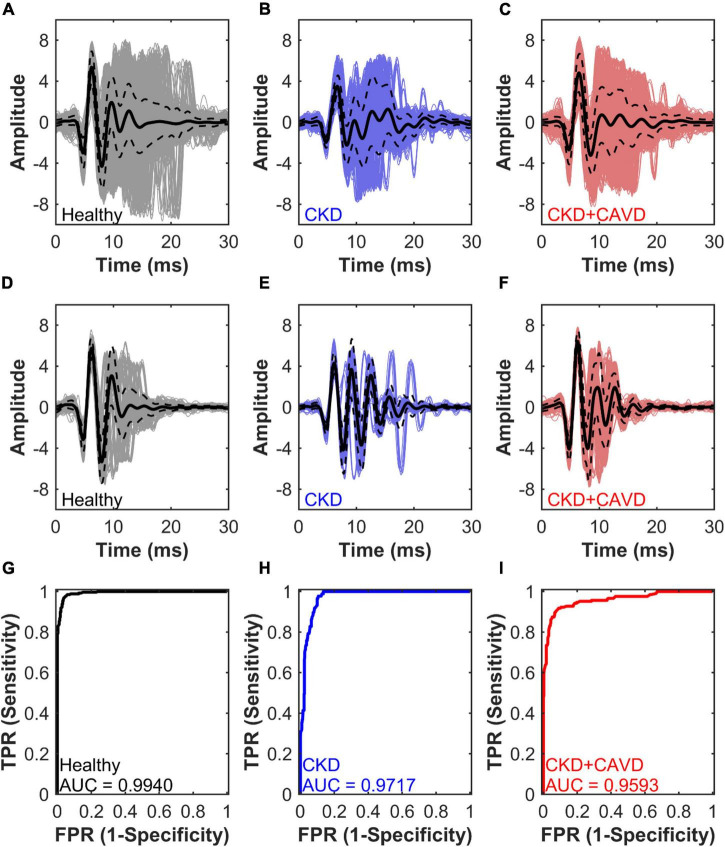
Algorithm performance for S2 sound classification. Average waveforms and algorithm performance of the final algorithm. Performance is measured by AUC, area under the receiver operating characteristic curve for testing set S2 sound classification. **(A–C)** Healthy, CKD, and CKD + CAVD training set average S2 sound waveforms. **(D–F)** Healthy, CKD, and CKD + CAVD testing set average S2 sound waveforms. **(G–I)** Healthy, CKD, and CKD + CAVD S2 sound classification AUC. TPR, true positive rate; FPR, false positive rate.

For our final algorithm, average classification error on withheld testing data (out-of-bag performance) was 5.98 ± 6.75%. The final algorithm training set included 19 mice and a total of 1,965 S2 sounds, with the breakdown per group as follows: Control (5M, 4F – 1,043 sounds), Adenine (3M – 254 sounds), and Adenine + HP (4M, 3F – 668 sounds). The average waveforms of all the S2 sounds per group are shown in Figures 7A–C. The algorithm used the variances of selected components as the inputs. The testing set included 5 mice and a total of 552 S2 sounds: Control (1M, 1F – 258 sounds), Adenine (1M – 87 sounds), and Adenine + HP (1M, 1F – 207 sounds). The average waveform for the S2 sounds correctly predicted by the algorithm per group is shown in Figures 7D–F. The Adenine + HP mice had a range of 0.27–39.03% AoV calcification per total leaflet volume. Average percentage of AoV calcification per total volume of the training and testing set in CKD + CAVD mice were 7.05 ± 0.55 and 17.06 ± 5.20%, respectively.

Figures 7D–F showed the performance of the final algorithm for the testing set. The AUC for identifying Healthy mice was the highest at 0.9940, followed by the AUC for identifying CKD at 0.9717 and AUC for CKD + CAVD at 0.9593 (Figures 7G–I). The confusion matrix (Table 2) compares the algorithms prediction performance with the true values. The accuracy for predicting healthy S2 sounds was 97.29%, with a negative predictive value of 95.80%. Accuracy for predicting CKD S2 sounds was 89.66%, with a PPV of 78.79%. Lastly, the accuracy for predicting CKD + CAVD S2 sounds was 84.54%, with a PPV of 95.62%. Of the 552 total S2 sounds in the testing set, 51.27% were classified as true positive, 45.47% as true negative, 1.27% as false positive, and 1.99% as false negative (Table 3).

**TABLE 2:** Algorithm’s confusion matrix.

	True values		
	Control (%)	Adenine (%)	Adenine + HP (%)
Predicted Values			
Healthy	97.29	0	2.71
CKD	0	89.66	10.34
CKD + CAVD	5.31	10.15	84.54

*Confusion matrix for algorithm developed to classify S2 sounds.*

**TABLE 3:** Algorithm’s predictive rate.

	Disease manifestation		
	Healthy (%)	CKD (%)	CKD + CAVD (%)
True positive (294 sounds, 51.27%)	0	31.08	69.72
True negative (251 sounds, 45.47%)	100	0	0
False positive (7 sounds, 1.27%)	100	0	0
False negative (11 sounds, 1.99%)	0	0	100

*Distribution of the algorithm’s classification accuracy and error.*

### Chronic Kidney Disease and Chronic Kidney Disease + Calcific Aortic Valve Disease Differentially Alter Aortic and Pulmonary Valve Function

**FIGURE 8:**
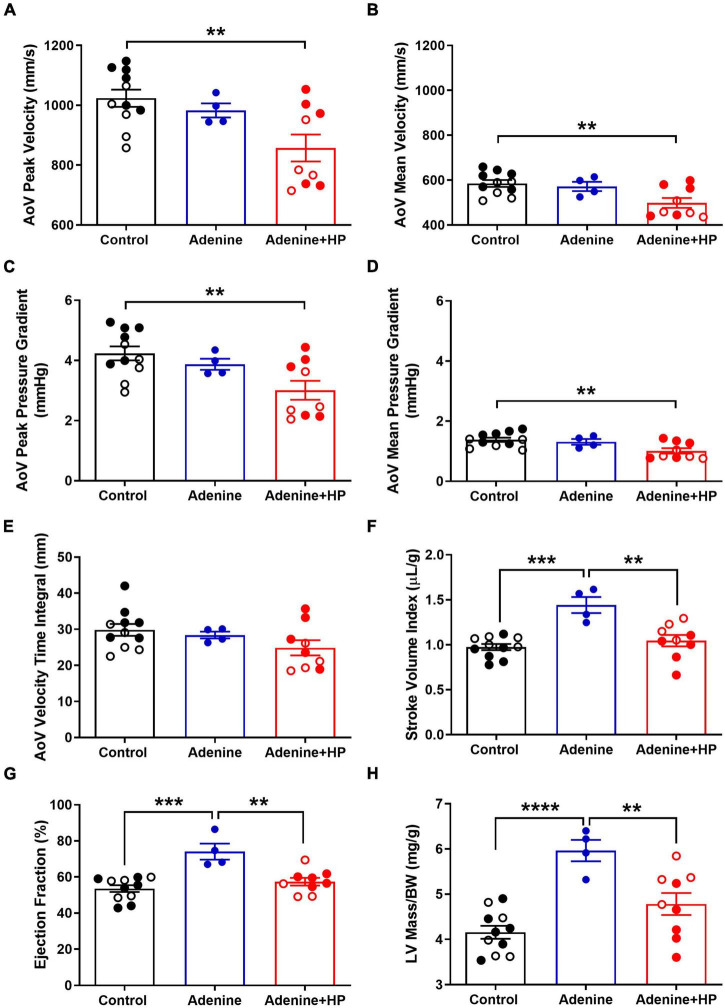
Echocardiographic measures of aortic valve and cardiac function. Aortic valve (AoV) and cardiac function assessed by echocardiography. AoV **(A)** peak velocity, **(B)** mean velocity, **(C)** peak pressure gradient, **(D)** mean pressure gradient, and **(E)** velocity time integral in Control, Adenine, and Adenine + HP mice. **(F)** Stroke volume, **(G)** ejection fraction, and **(H)** corrected LV mass in Control, Adenine, and Adenine + HP mice. One-way ANOVA with Tukey’s multiple comparisons test: *P < 0.05, **P < 0.01, ***P < 0.001, ****P < 0.0001. Male mice (●) and female mice (○). LV, left ventricle; BW, body weight.

**FIGURE 9:**
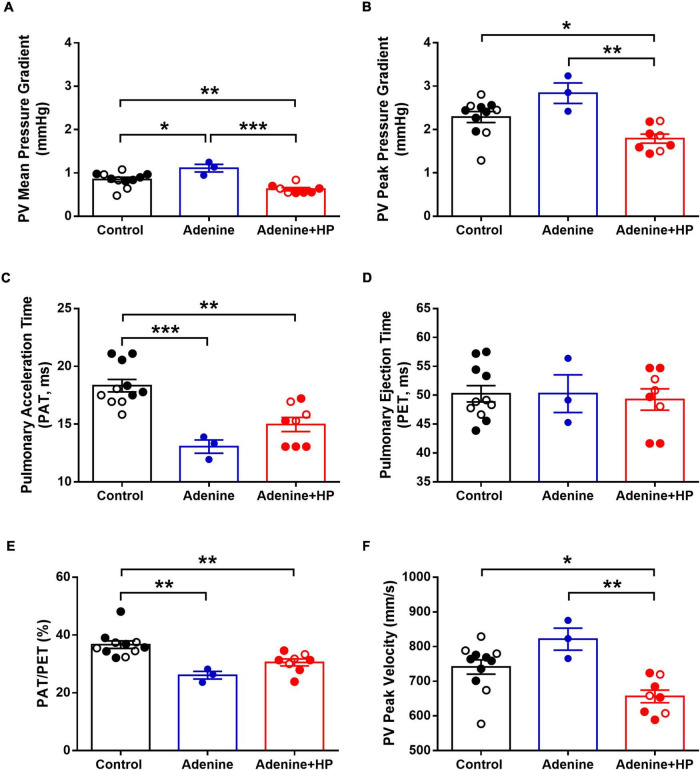
Echocardiographic measures of pulmonary valve function. Pulmonary valve (PV) function assessed by echocardiography. PV **(A)** mean pressure gradient, **(B)** peak pressure gradient, **(C)** pulmonary acceleration time, **(D)** pulmonary ejection time, **(E)** ratio of acceleration to ejection time, and **(F)** PV peak velocity in the Control, Adenine, and Adenine + HP mice. One-way ANOVA with Tukey’s multiple comparisons test: *P < 0.05, **P < 0.01, ***P < 0.001. Male mice (●) and female mice (○). PAT, pulmonary acceleration time; PET, pulmonary ejection time.

Analysis of echocardiographic data revealed that the peak and mean AoV velocity and pressure gradients were significantly (*P* < 0.01) decreased in the Adenine + HP group when compared to the Control group (Figures 8A–D). AoV velocity time integral was significantly lower in the CKD group only (Figure 8E). We did not observe a regurgitant jet in either the AoV or PV for any of our mice. Since the S2 sound is produced by closure of the AoV and PV, S2 sound analysis could be influenced by PV functional changes. Therefore, common hemodynamic measures that assess PV and right ventricular function were considered. There were significant changes in PV mean pressure gradient amongst all groups (Figure 9A). When compared to Control, there was a 1.31-fold increase of PV mean gradient in the Adenine group, but a 1.35-fold decrease in the Adenine + HP group. The Adenine + HP group also had significant PV functional changes when compared to Control and Adenine groups in the following parameters: peak pressure gradient, acceleration time (PAT), PAT/ejection time (PET) and peak velocity (Figures 9B–F).

### Chronic Kidney Disease Alters Left Ventricular Function

When classifying stages of aortic stenosis, the most common cardiac hemodynamic parameters to consider include stroke volume, LV ejection fraction and LV hypertrophy (19). Stroke volume index, expressed as the stroke volume normalized to body weight, was significantly higher in the Adenine group (1.44 ± 0.18 μL/g) when compared to the Control group (0.97 ± 0.03 μL/g) and the Adenine + HP group (1.05 ± 0.06 μL/g) (Figure 8F). The LV ejection fraction per group was the following: Healthy 53.57 ± 0.57%, Adenine 74.10 ± 2.23%, and Adenine + HP 57.42 ± 0.70% (Figure 8G). The Adenine group had significant changes in LV ejection fraction, with a 1.38- and 1.28-fold greater than the Control and Adenine + HP groups, respectively (Figure 8G). Normalized LV mass, as measured by echocardiography, was greater in the Adenine group (5.97 ± 0.04 mg/g) than in the Control (4.16 ± 0.12 mg/g) and Adenine + HP (4.78 ± 0.08 mg/g) groups (Figure 8H).

## Discussion

### The S2 Sound Identifies the Presence of Aortic Valve Calcification in Mice

In the present study, we found that the addition of high phosphate significantly induced AoV calcification in an existing mouse model of CKD with vascular calcification. Only the mice on the Adenine + HP diet exhibited AoV classification, with an average calcification per total AoV volume of 8.51 ± 6.71% per mouse. Regardless of AoV hemodynamics, our algorithm had a high predictive performance and could differentiate characteristics of S2 sounds from Healthy (Control) mice to those with CKD (Adenine) and CKD + CAVD (Adenine + HP). Echocardiogram parameters showed changes in AoV flow in the Adenine + HP group when compared to the Control group. The Adenine + HP had significantly lower AoV pressure gradients and velocities compared to the Control group (Figures 8A–D). Normalized LV mass in the Adenine + HP mice was similar to the Control mice, suggesting that these mice do not exhibit LV pressure overload due to AS. When compared to Control, EF was normal in the CKD + HP group and elevated in the CKD group. Therefore, some of the CKD + HP mice could be exhibiting echocardiogram measures indicative of low-flow, low-gradient AS with normal EF. However, none of the AoV flow parameters in CKD + CAVD mice had elevated mean pressure gradients, a characteristic of high-gradient AS. The algorithm was able to learn S2 sound features from a training set of mice and accurately predict 84.54% of sounds from new mice based on the presence of CKD-induced CAVD. More importantly, the algorithm identifies the potential of using the S2 sound to detect AoV calcification independently from AoV flow changes measured by echocardiogram.

Pulmonary valve structure and function could also influence S2 sound characteristics. There are only two PV diseases: stenosis and regurgitation. PV stenosis occurs due to PV calcification – however, calcification of the PV is almost exclusively caused by a congenital defect (31). During echocardiogram, we did not observe any regurgitant jet across the PV. Additionally, there are other cardiovascular diseases that could influence changes in the S2 sound, such as hypertensive heart disease and pulmonary hypertension. We did not observe high blood pressure in our Adenine nor Adenine + HP groups when compared to Control, thus reducing the likelihood of S2 sounds changes occurring due to hypertensive heart disease. Measures of pulmonary artery pressure *via* echocardiogram in mice are inaccurate since the formulas are based on human clinical equations (32). Researchers have found that pulmonary acceleration time (PAT) inversely correlates to increased catheter based measures right ventricular systolic pressure, which is indicative of pulmonary hypertension (33). PAT is decreased in both the Adenine and Adenine + HP groups when compared to Control, which could indicate an increase in pulmonary arterial pressure. However, in order for the algorithm to learn from a different disease (e.g., pulmonary hypertension), the labeling criteria would need to separate each mouse into the same disease categories of the current labeling criteria (Control as Healthy, Adenine as CKD, and Adenine + HP as CKD + HP). This means that to label each mouse into its corresponding disease category, there would have to be values of pulmonary hemodynamics that are significantly different amongst the groups and the values do not overlap with each other. While PV mean pressure gradient is significantly different amongst all groups (Figure 9A), individual mice from the different groups have similar values, but the algorithm still classifies these mice correctly. Therefore, it is likely that the differences in S2 sound characteristics in the CKD + CAVD group are due to the presence of AoV calcification.

### Accurate Classification of S2 Sounds From Chronic Kidney Disease Mice Could Be Explained by Markers of Heart Failure

The algorithm was also able to accurately classify S2 sounds from the CKD group, which suggests there may be other cardiac changes that influence the S2 sound in the CKD mouse model not associated with CAVD. The Adenine group had a significantly higher stroke volume index, LV ejection fraction, and LV mass when compared to the Control group. An increased or preserved ejection fraction has been associated with both systolic (34) and diastolic (35) dysfunction in non-CKD mouse models. An increase in stroke volume index and ejection fraction usually occurs due to volume overload, which commonly presents in dialysis patients and is an important prognostic factor in determining interventional outcome (36). About 50% of patients that develop heart failure with preserved ejection fraction also have CKD (37). Pulmonary hypertension is a marker of deteriorating heart failure and is common amongst CKD patients (38). As mentioned, the Adenine and Adenine + HP mice have lower PAT that could correlate to increased right ventricular systolic pressure (33). The PAT of Adenine mice is much lower than the mean and lowest value of PAT from the control group. If the S2 sound in CKD + CAVD mice is identifying the presence of AoV calcification, then the S2 sound in CKD mice must be detecting a different cardiac abnormality. Increased right ventricular pressure, a marker of advanced heart failure, could also lead to increased PV pressure and velocity. Therefore, we hypothesize that our algorithm accurately learned and differentiated features of the S2 sound from mice in the CKD group due to functional changes associated with advanced heart failure. The S2 sound in CKD + CAVD mice may be altered due to changes in the aortic component (A2) of the sound due to AoV calcification. The pulmonary component (P2) of the S2 sound in CKD mice may be altered due to a delay in closure of the PV due to increased right ventricular systolic pressure and pulmonary hypertension. Further studies are needed to identify the underlying cardiovascular pathophysiology that would cause detectable changes in the S2 sound of CKD mice without CAVD.

### S2 Sound Feature Classification Is Affected by Disease Severity

In this study, we found that the average amount of AoV calcification (by volume) from mice in the training and testing sets correlates to algorithm classification accuracy, which could be important in disease classification of the S2 sound. Variability in our model dependent on the subject-wise split can be explained by the different degrees of AoV calcification in our mice. The higher the average AoV calcification per total volume used in the training set, the lower the algorithm accuracy. This phenomenon may be explained by the evolution of S2 sounds over the course of CAVD. As CAVD progresses, subtle S2 features of disease may become more pronounced. Therefore, training the algorithm using the pronounced S2 features of heavily calcified leaflets may result in missed subtle features in the mice with lower levels of calcification. In contrast, training the algorithm with the subtle S2 features allows the model to identify both subtle and pronounced disease features. Future studies with more mice will test how different amounts of AoV calcification influence S2 features and assess if there must be a minimum amount of AoV calcification present to identify changes in these features.

Classification variability of CKD + CAVD and CKD mice may be due to different progression and stages of disease. As previously reported by Tani et al., the addition of high phosphate to the adenine diet (i.e., Adenine + HP group) improves renal function and stops the rapid loss of body weight that occurs after the diet is initiated (23). Despite causing significant AoV calcification, the addition of high phosphate to the Adenine diet seems to improve cardiac function of mice in the Adenine + HP group. Parameters of cardiac function (stroke volume index, ejection fraction, and LV mass) were similar in the Control and Adenine + HP groups (Figures 8F–H). Only the Adenine group had significant changes in cardiac function indicative of increased volume overload. Although the mice were administered the same diet for the same amount of time, differences in disease progression and manifestation are bound to exists in biological replicates. It is known that murmur and amplitude of S2 components vary due to severity of AS in humans. Therefore, changes due to cardiac disease severity could also occur in the CKD group. Future studies will assess cardiac function in a larger subset of CKD mice to determine cardiac abnormality responsible for S2 sound changes. A larger dataset that is more representative of the different disease severities and progression could reduce algorithm variability and improve disease classification.

### Manifestation of Cardiac Murmurs in Mice Could Be Indicators of Structural Heart Disease

In addition to detecting S2 sound changes in mice, we also detected cardiac murmurs. We found an S4 sound in some mice from the Adenine group and a systolic murmur (holosystolic in appearance) in mice from the Adenine + HP group. Systolic murmurs are usually caused by semilunar valve stenosis or atrioventricular valve regurgitation, while S4 sounds can be caused by a variety of cardiovascular issues (39). A pathologic S4 sound indicates reduced ventricular compliance, and is present in patients with systemic hypertension, AS, and hypertrophic cardiomyopathy (39). Both murmurs, however, were not detected in every cardiac cycle. In humans, the presence of cardiac murmurs not only depends on severity of disease, but also auscultation location, response to maneuver, respiration, presence of other cardiovascular comorbidities, and, most importantly, hemodynamic patterns. Inherently, mice have a much higher heart rate than humans. An increased heart rate leads to faster blood flow velocities, which could cause more turbulent flow in cardiac structures and, consequently, affect the presence of cardiac murmurs. We demonstrated the feasibility of using the S2 sound as a direct measure of AoV calcification, which is more reliable when assessing aortic stenosis than changes in hemodynamic patterns or presence of murmurs in humans (19).

### Valvular Structural and Functional Differences Due to Chronic Kidney Disease-Induced Aortic Valve Calcification

No previous reports exist on AoV calcification due to an Adenine + HP diet in mice. The observed variability in AoV calcification is also reported in other mouse models of CAVD. Only half of Western diet-fed apoE−/− mice, the most common mouse model used to study CAVD, develop AoV calcification when fed the diet for 9 months (40). Other transgenic mouse models with diet-induced CAVD have shown similar results, with only a percentage of mice developing calcific plaque over the course of 6–20 months (41, 42). Commonly, calcification in mouse AoV leaflets is quantified by imaging AoV cross-sections and comparing the intensity of a calcium tracer to the AoV area (43–45). It is difficult to quantify total AoV calcification since the AoV area needs to be manually selected for each cross-section. Additionally, AoV area errors can occur due to the difficulty of obtaining intact cross-sectional areas of the leaflet without disturbing its shape. In this article, we identified a new method of quantifying AoV calcification using whole-mount AoV leaflets. Additionally, though the range of calcification varied, we identified that all of our Adenine + HP mice exhibit AoV calcification within a 12-week regimen.

It is well established that CKD induces vascular and valvular calcification in different animal models (20, 46). However, little is known of AoV hemodynamic changes due to CKD + CAVD in mice. Our experimental groups showed that only the Adenine + HP diet leads to AoV calcification, but interestingly, we did not detect changes in common echocardiogram markers that suggest the presence of aortic stenosis in our mice. The most common flow-gradient pattern of aortic stenosis is increased AoV peak velocity and mean gradient (47), as observed in a mouse model of CAVD induced by AoV wire injury (48). The Adenine + HP group had lower AoV peak velocity and mean pressure gradient with preserved ejection fraction. In humans, a subset of patients with severe aortic stenosis present with preserved ejection fraction and low-flow, low-gradient (19), as observed in our mouse model. These patients do not present with the systolic murmur associated with aortic stenosis (49). Quantification of AoV calcification by CT imaging is a more accurate method to measure severity of aortic stenosis, especially in low-flow, low-gradient patients (19). However, AoV calcium score required to have significant CAVD measured *via* CT has not been translated to small animal models. While AoV calcium score positively correlates with aortic stenosis severity and has a high sensitivity (50), few studies have assessed the prognostic value of AoV calcium score. Further studies are needed to better understand how progression of AoV calcification affects cardiac function and identify optimal timing of intervention, which can be accomplished in humans or animal models of CAVD. New markers of aortic stenosis that correlate well with disease prognosis and are accessible to the general population are needed to improve disease diagnosis.

### Variability of Model Performance Occurs Due to Subject-Wise Split of Training and Testing Sets

In machine learning, there are two main approaches for splitting the data into training and testing sets: (1) record-wise split where each record (i.e., a single S2 sound) is randomly assigned to either the training or testing set or (2) subject-wise split where all records from a subject (i.e., mouse) are randomly assigned to either training or testing set. Although used extensively in digital health (12, 51, 52), record-wise splits causes identity confounding, where the classifier is learning from the identity of the subject rather than the identity of the disease (53). As a result, this leads to a higher AUC and a lower prediction error that overestimates the use-case performance of the diagnostic system. Therefore, we chose the subject-wise split method to avoid identity confounding and accurately assess the predictive performance of the S2 sound to identify new cases of CAVD.

We applied the leave-*p*-out cross-validation method to identify all the possible different ways that the mice could be split into training and testing sets and how this split affects the classifier’s performance. When splitting the data subject-wise, model underfitting can occur depending on how the subjects are split for training and testing sets. As observed in our model, the accuracy and misclassification error of the classifier varied based on which mice were assigned to the training or testing sets. Model underfitting and algorithm variability was caused by the low number of mice, which is a limitation of our study. However, model underfitting can be improved by increasing the number of subjects (i.e., number of mice per group). The rationale, as Chaibub Neto et al. explained, is that as the number of subjects in the training set increase, the classifier is learning more features related to disease that have a better chance of classifying unseen cases (53). To test the hypothesis that the S2 sound can detect the presence of CAVD, we identified the combination of mice (*p*) that yielded the lowest classification error and reported its performance. By doing this, we are ensuring that the training set contains sufficient information for each condition (Healthy, CKD, and CKD + CAVD) to accurately classify S2 sounds from new mice.

### Validity and Implications of Using Mouse Models to Study the Correlation Between Heart Sounds and Aortic Valve Disease

In this article, we showed that mouse heart sounds can be recorded using a commercially available digital stethoscope. Mice were anesthetized to ensure that heart sounds could be identified in the signal despite the presence of respiratory movement artifacts. It takes approximately 5–10 min to anesthetize mice and record heart sounds. In this time frame and with the concentration of isoflurane used, there should not be any alterations to cardiac hemodynamics due to the anesthetic (54). We also developed a new ROI-SSE segmentation method to segment heart sounds. We validated the ROI-SSE segmentation method by comparing it to the existing ECG segmentation method. The error between the two methods was low, with the ROI-SSE method identifying an average of 8 (out of 134) fewer cycles than the ECG method. An advantage of this method is that it does not require simultaneous recording of two biosignals, thus simplifying data acquisition.

Although studies have previously reported analyses of heart sounds in mice (55, 56), to our knowledge, none have reported on pathophysiological heart sound changes due to cardiovascular diseases. Mice, like humans, have 4-chamber hearts and the S1 and S2 sounds are caused by the closure of atrioventricular and semilunar valves, respectively. Valve closure, and consequently S1 and S2 sounds, is influenced by both mechanical properties of valvular tissues and transvalvular pressure gradients. Mouse models of CAVD have shown increased LV systolic pressure and transvalvular pressure gradients similar to humans (57, 58), which could suggest that the characteristics of the S2 sound are also similar. The extracellular matrix protein composition that drives gross mechanical properties of AoV leaflets remains the same in human and mouse. Both AoV tissues have the same basic organization and orientation with some differences in organization due to the larger size of human AoV leaflets (59). The difference in tensile properties of human and mouse AoV tissues, both healthy and diseased, remains unknown due to the small, microscopic size of mouse AoV leaflets. Recently, we developed a method to quantify equiaxial properties of mouse AoV leaflets (60), which could lead to a better understanding of the similarities between the mechanical properties of both species and how they influence sound production.

While the prevalence, pathophysiology, and significance of cardiac murmurs has been studied extensively in humans (61–63), auscultatory changes during early stages of disease have not. Early valve remodeling may precede gross functional changes seen in echocardiography, and thus would require more expensive or invasive diagnostics. Identifying whether the S2 sound can detect the presence of AoV calcification prior to functional remodeling changes that alter hemodynamics could improve understanding and progression of disease. The advantages of using mouse models to study CAVD include having the ability to control disease progression and a much lower financial cost when compared to humans. Most importantly, in the context of CAVD, mouse models allow us to study longitudinal changes of a disease in a relatively short period of time compared to the human counterpart. Therefore, more studies are needed to identify the correlation between heart sound characteristics and heart disease in mice. Future studies with a larger number of animals and different mouse models of CAVD could: (1) validate the feasibility of using the S2 sound to detect early stages of CAVD and (2) warrant further investigation to determine whether changes in S2 sound characteristics of mice also occurs human.

## Conclusion

New, low-cost diagnostic techniques that reduce the importance of symptom reporting and auscultatory skills are needed to improve aortic stenosis diagnosis, especially in underserved populations. In this study, we hypothesized that structural changes due to AoV calcification could be found in the S2 sound. We built an ensemble learning-based algorithm that could accurately classify mouse S2 sounds based on the presence of AoV calcification independent of hemodynamic changes measured *via* echocardiogram. The findings in this study highlight the potential of using the S2 sound as a new method of identifying AoV calcification.

## Data Availability Statement

The raw data supporting the conclusions of this article will be made available by the authors, without undue reservation.

## Ethics Statement

The animal study was reviewed and approved by the Florida International University – IACUC Protocol 20-045.

## Author Contributions

VD developed the methodology and conducted the phonocardiogram signal acquisition and analysis. HN contributed to the animal work and statistical analysis. VD and HN developed the original draft of the manuscript. HN and SN contributed to the confocal image acquisition. DC analyzed the confocal images. SS, CI, and LS contributed to echocardiography data acquisition and analysis. ZD and AB have contributed to data analysis methodology. JH was the senior and last author and formulated the original research idea and aided in experimental design, data analysis, and manuscript development. All authors provided critical feedback of manuscript drafts.

## Conflict of Interest

The authors declare that the research was conducted in the absence of any commercial or financial relationships that could be construed as a potential conflict of interest.

## Publisher’s Note

All claims expressed in this article are solely those of the authors and do not necessarily represent those of their affiliated organizations, or those of the publisher, the editors and the reviewers. Any product that may be evaluated in this article, or claim that may be made by its manufacturer, is not guaranteed or endorsed by the publisher.

## Nomenclature

### Resource Identification Initiative

- Phonocardiogram signal acquisition and analysis software (MATLAB, RRID:SCR_001622).
- Tail cuff system (Kent Scientific Coda Blood Pressure System, RRID:SCR_018585).
- Confocal microscope (Olympus BX61 Upright Wide Field Microscope, RRID:SCR_020343).
- Image analysis software (National Center for Microscopy and Imaging Research: ImageJ Mosaic Plug-ins, RRID:SCR_001935).
